# Institutional Effects on Early Palliative Care among Mexican-Heritage Elders

**DOI:** 10.1093/geroni/igab046.3231

**Published:** 2021-12-17

**Authors:** Susan Miller, Ester Carolina Apesoa-Varano

**Affiliations:** 1 UC Davis; 2 UC Davis, Sacramento, California, United States

## Abstract

This paper addresses Mexican-heritage older people’s experiences with early palliative care (EPC). EPC is the early provision of medical, social and spiritual reports to relieve suffering. Empirically, Mexican-heritage older people are known to have less access to EPC and, when they access it, to receive care of lower quality. However, little work has explored how Mexican-heritage older people think about and access such care. The paper addresses this gap. Methods are longitudinal: 36 Mexican-heritage people ranging in age from 55 to 90 years completed longitudinal semi-structured qualitative interviews, for a total of 69 interviews. Results explore how respondents’ participation in social institutions may mediate the effects of larger social structural constraints on their health and access to care.

